# Agreement between Keratometric readings by VERION image guided System, Galilei G4 and Pentacam

**DOI:** 10.12669/pjms.343.14577

**Published:** 2018

**Authors:** Asad habib, Muhammad Saim Khan, Mazhar Ishaq, Muhammad Amer Yaqub

**Affiliations:** 1 Dr. Asad Habib, MBBS. Department of Ophthalmology, Armed forces institute of Ophthalmology (AFIO), Rawalpindi, Pakistan; 2 Dr. Muhammad Saim Khan, FCPS, FICO, MRCSEd. Department of Ophthalmology, Armed forces institute of Ophthalmology (AFIO), Rawalpindi, Pakistan; 3 Dr. Mazhar Ishaq, FCPS/FRCSEd/FRCOphth. Department of Ophthalmology, Armed forces institute of Ophthalmology (AFIO), Rawalpindi, Pakistan; 4 Dr. Muhammad Amer Yaqub, MCPS, FCPS, FRCS.Ed. Department of Ophthalmology, Armed forces institute of Ophthalmology (AFIO), Rawalpindi, Pakistan

**Keywords:** Keratometry, Verion optical imaging, Pentacam, Galilei

## Abstract

**Objective:**

To study Agreement between Keratometric readings by VERION image guided System, Galilei G4 and Pentacam.

**Methods:**

The quasi experimental study was conducted at Armed Forces Institute of Ophthalmology, Rawalpindi, Pakistan from August 2016 to December 2016. Twenty five patients fulfilling the inclusion criteria participated in the study. All Patients were subjected to Keratometric assessment using Galilei G4 Dual Scheimpflug analyzer (Ziemer, Switzerland), Wavelight Oculyzer II (Pentacam, Germany) and Verion image guided system (Alcon). Steep and flat meridian and diopter of astigmatism by three systems were recorded and endorsed. All readings were taken by the same observer. Statistical Program for Social Sciences (SPSS) version 22.0 was used for statistical analysis. Results analyzed for significance by t-tests and Interclass correlation analysis. In t tests, P values of <0.05 was considered statistically significant while interclass coefficient of >0.7 was considered acceptable.

**Results:**

Fifty eyes of twenty-five patients (22 male, 28 female) with mean age of 29.50 ± 3.46 years were studied. Flat K, steep K and dioptric power of astigmatism were measured with verion, pentacam and Galilei G4. Interclass correlation analysis showed agreement between individual variables measured by the three devices, while one sample t test showed no significant difference between dioptric power of astigmatism between Verion-Pentacam and Verion- Galilei group. (p 0.178 for former and 0.622 for later group).

**Conclusion:**

Verion image guided system is comparable to other instruments used currently for keratometry. Verion can be interchangeably used with Pentacam and Galilee G4 in assessing corneal astigmatism.

## INTRODUCTION

Keratometry is the assessment of corneal surface powers. Different power in different meridian cause astigmatism. Corneal surface power is crucial in determining power of IOL^1^ required in cataract surgeries and planning of refractive surgeries.^2^,^3^

VERION image guided system is a collection of reference, planning units and digital marker to access, plan and guide the surgeon throughout surgical procedure aiming to minimize the post op residual astigmatism. Accessing the steep and flat meridians and planning incision sites and finally using digital marker to help make incisions improve the overall outcome of cataract surgery. VERION is studied to be efficient in reducing the residual astigmatism after cataract surgery.^4^ VERION measures Keratometry and various other parameters by taking a high resolution photograph of patient’s eye.

Pentacam and Galilei G4 are non-contact devices which make 2-D and 3-D images of anterior segment, measures corneal topography and pachymetry. Pentacam uses combination of slit illumination system and a rotating Scheimpflug camera to construct topographic images of anterior chamber of eye while the later uses a double Scheimpflug camera and a Placido topography system. Pentacam has been studied in comparison with other instruments.^5^,^6^,^7^ Various studies have assessed the repeatability and precision of corneal power measurements by various available instruments.^8^,^9^ The efficacy of VERION image guided system for taking the Keratometric reading of cornea has not been studied vastly. One study compared Verion Optical Imaging System, autokeratometer, IOLMaster and Pentacam suggesting no significant difference between them.^10^

Armed Forces Institute of ophthalmology is a tertiary care hospital and has recently acquired VERION image guided system. The rationale of conducting this study is to access the efficacy of VERION image guided system in accessing corneal power and astigmatism as compared with other Keratometry devices in our population.

## METHODS

The study was a Quasi Experimental and conducted in Armed Forces Institute of Ophthalmology from August to December 2016 on 50 eyes. Non-probability (purposive) sampling technique was used. Both male and female patients with no history of prior cataract or refractive surgery were included. Patient with history of corneal dystrophies, ocular trauma, previous ocular surgery, glaucoma, diabetes, corneal ecstasies, systemic diseases (such as collagen vascular diseases) and Contact lens wearers in last two week were excluded from study. After approval by the Ethical Committee informed consent was taken from all the patients prior to inclusion in the study. All Patients were subjected to keratometric assessment using Galilei G4 Dual Scheimpflug analyzer (Ziemer, Switzerland), Wavelight Oculyzer II (Pentacam, Germany) and Verion image guided system (Alcon). Three consecutive readings were taken by each device and average readings were recorded. Steep and flat meridian and diopter of astigmatism by three systems were compared.

Statistical Package for Social Sciences (SPSS 22.0) for windows was used for comparative analysis. The continuous data was described in terms of mean ±SD (Standard deviation) while categorical data was depicted in frequencies for each group. Results analyzed for significance by t-tests and Interclass correlation analysis. In t tests, P values of <0.05 was considered statistically significant while interclass coefficient of > 0.7 was considered acceptable.

## RESULTS

Fifty eyes of 25 patients (22 male, 28 female) were included in the study and each eye of the patients was considered separately. Age of the patients ranged from 23 to 36 years with a mean of 29.50 + 3.46 years (Table-I). Mean Steep K reading taken by Pentacam, Verion and Galilei were 44.64 + 1.89, 44.41 + 1.82 and 44.60 + 1.78 respectively. While mean astigmatism was 1.77 + 1.18, 1.87+1.23, and 1.86+ 1.14 by Pentacam, Verion and Galilei G4 respectively. Interclass correlation analysis was done which showed reliable measurements of flat K, steep K and astigmatism by the three devices. (Table-II) One sample t test was then performed to assess the mean difference and significance levels between astigmatism measurements by Verion and Pentacam as well as Verion and Galilei G4. Results showed that the difference was not significant and the devices can be used interchangeably.

**Table-I:** Descriptive statistics.

	N	Minimum	Maximum	Mean	Std. Deviation
Age	50	23	36	29.52	3.466
SteepK Pentacam	50	42.80	50.10	44.6440	1.89491
SteepK Verion	50	42.51	49.82	44.4136	1.82025
SteepK Galilae	50	42.53	49.19	44.6086	1.77939
Astigmatism pentacam	50	.60	4.60	1.7720	1.18495
Astigmatism Verion	50	.40	4.81	1.8736	1.23926
Astigmatism Galilae	50	.63	4.87	1.8464	1.14867

**Table-II:** Intraclass Correlation Coefficient (95% CI).

	Verion	Galilee	Pentacam
*Flat K*			
Verion	-	0.914	0.940
Galilee	0.914	-	0.802
pentacam	0.940	0.802	-
*Steep K*			
Verion	-	0.926	0.917
Galilee	0.926	-	0.768
pentacam	0.917	0.768	-
*Astigmatism*			
Verion	-	0.952	0.979
Galilee	0.952	-	0.975
pentacam	0.979	0.975	-

## DISCUSSION

Keratometry has its implications in assessment of anterior segment diseases^11^ as well as intraocular lens power calculation before cataract surgery.^12^ It has been a source of error in calculation of Intraocular Lens (IOL) power in the past. The present study compares keratometric reading and astigmatism measured by verion image guided system with Pentacam and Galilei. All use corneal radii of curvature and keratometric index but make use of different technologies. The measurements are considered in agreement if the difference between them is not more than a specified limit (significance level).

Over the years many devices have been developed to access corneal shape and curvature, starting from the manual keratometers, to automated ones and infrared based and 3D topographers which can access both anterior and posterior corneal surfaces, create corneal thickness maps and AC depth analysis as well as IOL power calculation. Keratomertry is a vital variable in IOL power calculation formulas. Different surgeons use different set of pre op investigations to get a good post op result. With the advent of new devices patients expectations have also gone high.

Verion image guided system is a new device that uses central 2.8 mm of cornea and refractive index of 1.3375.^13^ There are three Infrared and 12 white lights that help in measurement of spherical power and astigmatism/cylinder power respectively. Verion consists of a reference unit and a planning software. Reference unit takes pre-operative photographs of eye and uses limbus, scleral vessels and iris to auto register the eye intraoperatively. Planning software plans the location of incisions according to target post op refraction. Intraoperative digital marker shows incision locations on screen during surgery for assistance of surgeon. Pentacam uses rotational Scheimpflug camera to measure corneal radii of curvature using central 4 mm zone. Galilee G4 has a dual Scheimpflug analyser. All the devices were calibrated according to the company’s recommendations prior to inclusion in the study to minimize any errors due to calibration problems.

**Table-III:** Mean Difference in astigmatism between Verion and other devices: One sample t test.

	Verion (mean difference)	Sig.
Pentacam	0.0480±0.248	0.178
Galilee	0.0264±0.37633	0.622

Our results are consistent with other studies^13^,^14^, showing that these three devices can be interchangeably used in different clinical settings and requirements.

Verion image guided system has got in it integrated digital marking system which helps in incision placement at any desired location e.g. at steep corneal axis or correct alignment of Toric IOL axis. Our study shows that there is generally no statistically significant difference between kertatometric readings taken by verion and other devices. Other Studies have included more parameters like mean K, and axis of astigmatism as well^15^,^16^ but we in our study have limited ourselves to steep K and dioptric power of astigmatism only.

VERION with its digital marking system helps surgeon plan surgical incision in order to target a zero post-operative astigmatism. What this study doesn’t add is how Verion helps in setting of Toric IOL placement and its usage in Femtolaser assisted cataract surgeries. This is not the mandate of our study. Further study needs to be done in this regard.

## CONCLUSION

Keratometric reading taken by VERION image guided system is in agreement with Pentacam and Galilei G4. Three devices can be used interchangeably in different clinical settings.

### Author’s Contribution

**AH:** Data collection, statistical analysis & manuscript writing.

**MSK:** Data collection and editing of manuscript.

**MI:** Basic idea, surgeon and final approval of version to be published.

**MAY:** Basic idea and final approval of version to be published.
